# Advantages and Limitations of Clinical Scores for Donation After Circulatory Death Liver Transplantation

**DOI:** 10.3389/fsurg.2021.808733

**Published:** 2022-01-05

**Authors:** Raphael P. H. Meier, Yvonne Kelly, Seiji Yamaguchi, Hillary J. Braun, Tyler Lunow-Luke, Dieter Adelmann, Claus Niemann, Daniel G. Maluf, Zachary C. Dietch, Peter G. Stock, Sang-Mo Kang, Sandy Feng, Andrew M. Posselt, James M. Gardner, Shareef M. Syed, Ryutaro Hirose, Chris E. Freise, Nancy L. Ascher, John P. Roberts, Garrett R. Roll

**Affiliations:** ^1^Division of Transplant Surgery, Department of Surgery, University of California, San Francisco, San Francisco, CA, United States; ^2^Department of Surgery, University of Maryland, Baltimore, MD, United States; ^3^Department of Anesthesia, University of California, San Francisco, San Francisco, CA, United States

**Keywords:** liver transplantation, donation after circulatory death, warm ischemia time, hepatectomy time, biliary complications, ischemic cholangiopathy, score

## Abstract

**Background:** Scoring systems have been proposed to select donation after circulatory death (DCD) donors and recipients for liver transplantation (LT). We hypothesized that complex scoring systems derived in large datasets might not predict outcomes locally.

**Methods:** Based on 1-year DCD-LT graft survival predictors in multivariate logistic regression models, we designed, validated, and compared a simple index using the University of California, San Francisco (UCSF) cohort (*n* = 136) and a universal-comprehensive (UC)-DCD score using the United Network for Organ Sharing (UNOS) cohort (*n* = 5,792) to previously published DCD scoring systems.

**Results:** The total warm ischemia time (WIT)-index included donor WIT (dWIT) and hepatectomy time (dHep). The UC-DCD score included dWIT, dHep, recipient on mechanical ventilation, transjugular-intrahepatic-portosystemic-shunt, cause of liver disease, model for end-stage liver disease, body mass index, donor/recipient age, and cold ischemia time. In the UNOS cohort, the UC-score outperformed all previously published scores in predicting DCD-LT graft survival (AUC: 0.635 *vs*. ≤0.562). In the UCSF cohort, the total WIT index successfully stratified survival and biliary complications, whereas other scores did not.

**Conclusion:** DCD risk scores generated in large cohorts provide general guidance for safe recipient/donor selection, but they must be tailored based on non-/partially-modifiable local circumstances to expand DCD utilization.

## Introduction

Despite an increased risk of recipient morbidity and mortality, donation after circulatory death (DCD) donors are increasingly used for liver transplantation (LT) to address the organ shortage and waitlist mortality (1). The majority of DCD liver utilization occurs at a relatively small number of centers in the United States (2). This is similar to practice patterns in many countries around the world, including the United Kingdom (UK), where much about DCD liver transplantation has been learned. Descriptions of outcomes and risk factors for non-anastomotic strictures, primary non-function, and graft failure have been identified by a number of DCD risk scores based on data derived from these high-volume centers (3–5). Many of the postulated risk factors and organ availability issues limiting liver transplantation vary dramatically between regions of the world and are non-/partially-modifiable, such as societal/cultural restrictions on donor intervention and management, the frequency of donor co-morbid conditions, preservation time related to organ transportation, liver allocation policy, availability of living donors, waitlist mortality, and Model for End-Stage Liver Disease-Sodium (MELD) score at the time of transplant. Therefore, existing risk scores may not be generalizable, and relying on them may risk the loss of transplant opportunities due to relatively unique local circumstances. Specifically, characteristics currently considered to be significant risk factors, such as donor body mass index (BMI) >25, cold ischemia time (CIT) >6 h, and recipient MELD >25, could limit local DCD-LT for some centers. We sought to determine differences in key outcome predictors and designed one simple DCD score derived from our local data and one complex DCD score derived from United Network for Organ Sharing (UNOS) data. We compared the ability of these to predict graft survival against existing complex scoring systems, including the UK- (5), University of California Los Angeles (UCLA)- (3), King's College Hospital (KCH)-DCD scores (4).

## Methods

### Study Design and Patients

**Figure 1:**
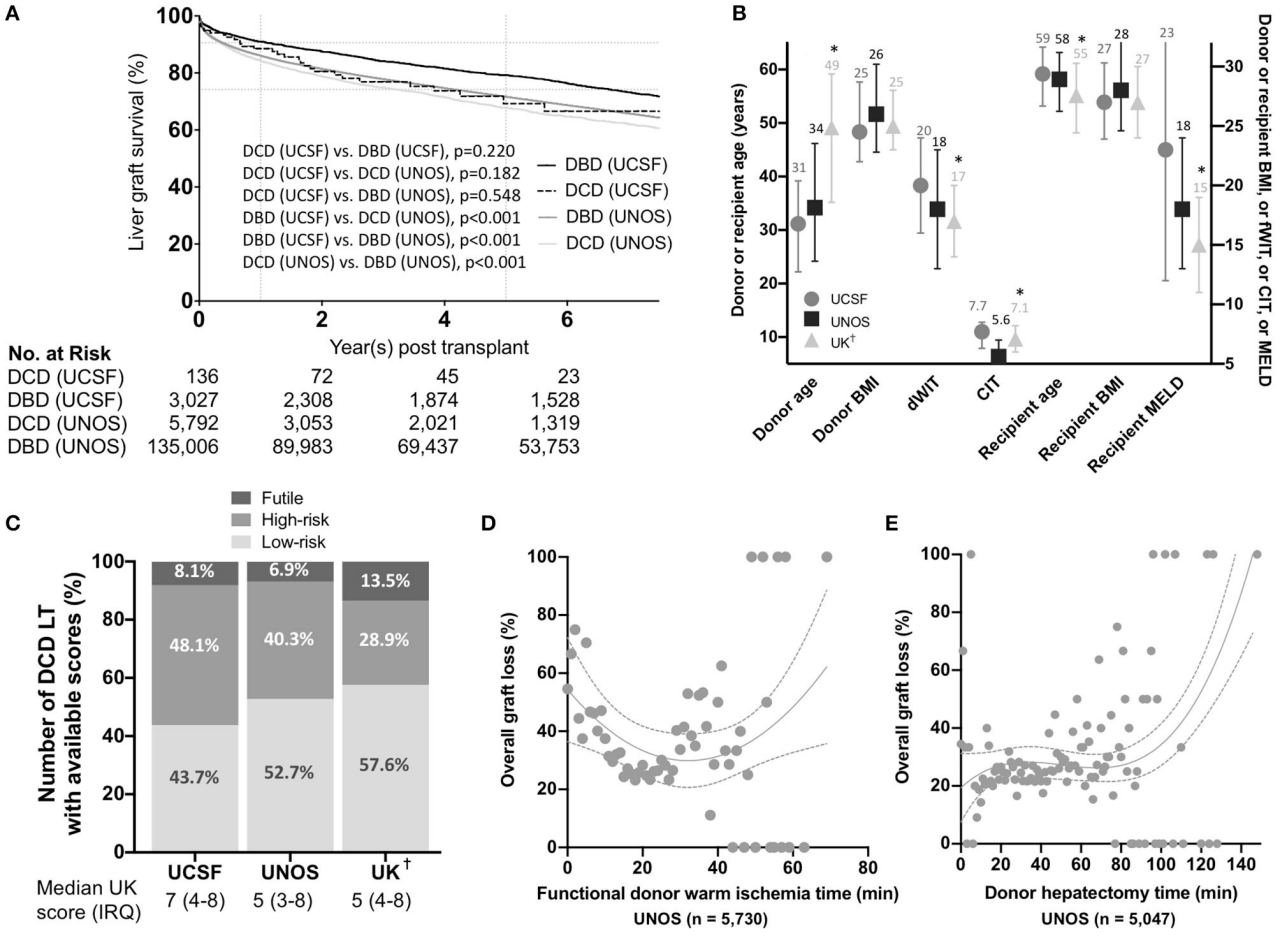
**(A)** Overall liver graft survival. Kaplan-Meier survival curves showing liver graft survival for donation after circulatory death (DCD) and donation after brain death (DBD) liver transplantation in the University of California, San Francisco (UCSF) and United Network for Organ Sharing (UNOS) cohorts. P-values were calculated using the Log-Rank test. Key variables and score values in the different US and UK cohorts. **(B)** Donor and recipient variables in the University of California, San Francisco (UCSF), United Network for Organ Sharing (UNOS), and United Kingdom (UK) cohorts. Median and interquartile ranges are shown. *P*-values were estimated using two-way ANOVA tests assuming a normal distribution. *denote a *p* < 0.05 compared to the UCSF group. **(C)** Percentages of donation after circulatory death (DCD) liver transplants (LT) in each category (i.e. low-risk, high-risk and futile) in the UCSF, UNOS, and UK cohorts (Percentages were calculated excluding missing score data to obtain a visual comparison between the three groups). Variation in overall graft loss along with donor warm ischemia time **(D)** and donor hepatectomy time **(E)**. Interpolations were performed using third-order polynomial equations. BMI, Body mass indexI; dWIT, Donor warm ischemia time; CIT, Cold ischemia time; MELD, Model for End-Stage Liver Disease-Sodium. As reported by Schlegel et al. (5)

Approval was obtained from the Institutional Review Board of the University of California, San Francisco (IRB 15-18341). Donor and recipient data for all consecutive adult (i.e., ≥18 yo) DCD liver transplants performed between 2005 and 2020 at the University of California, San Francisco (UCSF) Medical Center and within the UNOS were extracted (*n* = 5,792). Liver graft survival using donation after brain death donors (DBD) from both cohorts was used for comparison in Figure 1A. Liver graft failure was defined as death or retransplantation. Median (interquartile range [IQR]) follow-up was 2.2 (1.0–4.6) years in the UCSF cohort and 2.3 (0.5–5.8) in the UNOS cohort. For the UCSF cohort, non-anastomotic strictures (NAS) were defined by the presence of intrahepatic strictures and dilatations related to ischemic cholangiopathy, occurring in the absence of ductopenic rejection or recurrent primary sclerosing cholangitis. NAS was diagnosed on endoscopic retrograde cholangiopancreatography (ERCP) or magnetic resonance imaging. Anastomotic strictures (AS) and biliary leaks were identified by ERCP or cross-sectional imaging. In order to compare the performance of a locally developed score *vs*. scores developed from a national database, we designed an index based on liver graft survival predictors in our local UCSF cohort (total warm ischemia time (WIT)-index), and a more complex universal-comprehensive (UC)-DCD score based on predictors from the UNOS cohort. We compared those newly developed scores with preexisting scoring systems, including the UK- (5), UCLA- (3), and KCH-DCD scores (4). The detail of the variables included in each score is available in the results section and was adapted from what was previously published (5), adding variables from the newly developed scores.

### Patient Selection and Organ Allocation

Patients diagnosed with end-stage liver disease were evaluated for candidacy by a multidisciplinary team and placed on the transplant waiting list (6). From 2002 to present, liver allocation has been according to MELD scores (7). Organ selection and LT were performed as previously described (6).

### Donor Selection and Procurement

DCD donor selection at UCSF was based on several criteria. Generally, donors were younger than 60 years, and a donor warm ischemia time (WIT) ≤30 min was strongly preferred (see *Definition of ischemia times* here-below). Intraoperative assessment of liver grafts was performed by the donor surgeon, and significant steatosis was avoided. Donor BMI, vasopressor requirement, and length of ICU stay have generally not been limiting variables. Organ transportation logistics to our center (we use airplane travel up to 50% of the time) largely dictate preservation times and have resulted in the large majority transplants with CIT ≥6 h (84%, 2,542/3,027 in DBDs and 80%, 109/136 in DCDs). Therefore, CIT ≥6 h were tolerated, although considerable efforts were expended to minimize CIT. Recipients requiring retransplant were generally not considered for DCD-LT. DCD donors were all Maastricht class III. Briefly, heparin was administered prior to mechanical ventilation withdrawal, typically outside of the operating room. When asystole occurred, the donor was transported to the operating room. An independent physician provided end-of-life care and was responsible for the declaration of death. After a 5-min mandatory waiting period, using the “super-rapid technique” (8), a midline incision was made, and both the right iliac artery and the inferior mesenteric vein were cannulated and flushed with 4L and 2L of cold University of Wisconsin solution, respectively. The liver and head of the pancreas were resected *en bloc* and flushed on the backtable with 1L of University of Wisconsin (hepatic artery and portal vein). LT was performed as previously described (6), typically utilizing the piggyback technique.

### Definition of Ischemia Times

Donor WIT (dWIT) is defined as the interval from the start of the agonal phase to aortic flush, per UNOS definition. In the UNOS data, the agonal phase starts when the donor's systolic blood pressure (SBP) drops below 80 mmHg and/or the donor SaO2 drops below 80% (9, 10). WIT times in function of previously published agonal phase start definitions (3–5) are available in Supplementary Figure 1. Donor hepatectomy time (dHep) is defined as the interval from aortic flush to the placement of the liver in 4°C cold storage solution. Graft CIT was defined as the interval from cross clamp to liver implantation in the recipient. dHep was the only variable with more than 10% missing data (12.9% in the UNOS cohort). Those missing and not missing data did not differ significantly on any of the characteristics examined except for the transplant era. The total WIT index was calculated using dWIT and dHep. All livers in our cohort were preserved with static cold storage.

### Statistical Analysis

Continuous variables were expressed as means and standard deviations (SD) and/or median [interquartile range (IRQ)] unless otherwise specified, and categorical variables were expressed as counts and percentages. Uni-/multivariable Cox proportional hazards regression was used to compute hazard ratios (HRs) and ninety-five percent confidence intervals (95% CI). Potential predictors with unadjusted *p* < 0.35 were considered for inclusion in the multivariable analysis.

Logistic regression was used for scores computation. The following candidate predictors were included: donor dWIT, dHep, cause of death, recipient/donor age, race, sex and BMI, cold ischemia time, recipient diabetes, dialysis, underlying liver disease, MELD, ascites, transjugular intrahepatic portosystemic shunt (TIPS), and the use of mechanical ventilation. Thresholds were based on previously published data (3–5). Thresholds were adjusted using multiple testing with one-unit increments to obtain the highest odds ratio (OR) associated with 1-year survival for individual predictors separately in the UCSF and UNOS cohorts. A dichotomy was used instead of a trichotomy and vice versa to obtain the highest OR and adjusted to obtain clinically meaningful categories. Potential 1-year graft survival predictors with unadjusted *p* < 0.1 were considered for inclusion in the multivariable model. A predictor with a *p* < 0.2 in the multivariate model was included in the final score. Score points were attributed proportionally to ORs (4, 11). Multiplication factors were determined in order to allow sufficient granularity, and a ±1-point adjustment was tolerated in order to obtain meaningful categories. For potential predictors with data missing for >10% of participants, we tested for differences in characteristics between the group missing data and the group with data available. A cohort of 825 DCD LT recipients from the UNOS dataset excluding region 5 (therefore not used for the score development) was used as a validation cohort for the total WIT index. We used DCD LT recipients with a dWIT>20 min, and dHep>30 min since no impact on survival is expected for variations below that threshold. A cohort of 248 DCD LT recipients extracted from the UNOS cohort performed between 2002 and 2004 (therefore not used for the score development) was used as a validation cohort for the UC-DCD score (no other DCD LTs were available for validation). Receiver operating characteristic (ROC) curves and c-statistics/area under the curve (AUC) were used to analyze score performance. For sensitivity analyses, multiple imputation using the Markov Chain Monte Carlo method with ten imputations was implemented for variables with data missing for >10% of participants.

Kaplan-Meier survival plots stratified by three categories of risk scores were generated. Model performance was evaluated using logistic regression concordance statistics (c-statistics) for survival data and calibration plots. A c-statistic of 1 corresponds to perfect discrimination, whereas a value of 0.5 corresponds to no discrimination ability. Patients missing one or more items to calculate a given DCD score were censored for survival analysis and score development.

Fisher's exact or chi-square tests, *t*-tests, and log-rank tests were used as appropriate. Hypothesis tests were two-sided, and the significance threshold was set to 0.05. Statistical analyses were performed using IBM SPSS Statistics version 26 (IBM Corp. Armonk, NY), SAS (Version 9.4, SAS Institute Inc., Cary, NC), and PRISM (Version 8, GraphPad Software, San Diego, CA). Ninety-five percent confidence intervals (95% CI) were reported, and an exact two-sided *p* < 0.05 was considered statistically significant.

## Results

### Baseline Characteristics and Survival

We included 136 adult DCD-LTs from the UCSF cohort and 5,792 DCD-LTs from the UNOS cohort. Donor and recipient characteristics are shown in Table 1. Donor median (IRQ) age was 31 (23–40) years in the UCSF cohort and 34 (24–46) in the UNOS cohort. The mean ± SD CIT was long in the UCSF cohort: 7.8 ± 2.5 h, compared to 6.0 ± 2.0 h in the UNOS cohort. Recipient median (IRQ) age was 59 (53–64) years and 58 (52–63) years in the UCSF and UNOS cohort, respectively. The median MELD score was high in the UCSF cohort: 23 (12–33), compared to 18 (13–24) in the UNOS cohort. The mean ± SD dWIT was 20.4 ± 5.9 min in the USCF cohort and 18.2 ± 7.4 min in the UNOS cohort. Times from extubation and incision to flush are available for the UCSF cohort in Supplementary Table 1. dHep was 40.5 ± 16.2 min and 34.2 ± 15.3 min in the UCSF and UNOS cohort, respectively. Liver graft survival for DCD and DBD are shown for the UCSF and UNOS cohorts in Figure 1A. One-year graft survival was 88.5% (DCD-LT UCSF), 91.0% (DBD-LT UCSF), 82.3% (DCD-LT UNOS) and 86.0% (DBD-LT UNOS). Five-year graft survival were 69.3% (DCD-LT UCSF), 79.1% (DBD-LT UCSF), 65.7% (DCD-LT UNOS) and 71.6% (DBD-LT UNOS). In the UCSF cohort, 4.4% (6/136) of the recipients had hepatic artery complications requiring intervention (thrombosis or stenosis), and 2.2% (3/136) of recipients had primary non-function; all three were retransplanted within 9 days. Three recipients (2.2%, 3/136) died within 2 weeks after transplantation.

**Table 1:** Recipient and donor baseline characteristics of donation after cardiac death liver transplantation.

**Characteristics**		**DCD LT** **(UCSF)** **(***n*** = 136)**	**DCD LT** **(UNOS)** **(***n*** = 5,792)**
**Recipient**			
Age at transplant, years	Mean ± SD	57.5 ± 9.0	56.5 ± 9.5
	Median (IRQ)	59 (53–64)	58 (52–63)
Male (%)		101 (74.3)	3,984 (68.8)
Pre-transplant BMI (kg/m^2^)	Mean ± SD	27.5 ± 5.3	28.6 ± 5.6
	Median (IRQ)	27.0 (23.9–30.3)	28.0 (24.6–32.1)
Race/ethnicity	- White - African American or Black - Hispanic - Asian - Hawaii - American Indian - Multiracial	74 (54.4) 4 (2.9) 37 (27.2) 18 (13.2) 1 (0.7) 2 (1.5) 0 (0)	4,321 (74.6) 435 (7.5) 734 (12.7) 204 (3.5) 10 (0.2) 54 (0.9) 34 (0.6)
Etiology	- HCV - HBV - EtOH - NASH/Cryptogenic - Genetic/metabolic - Autoimmune/PBC/PSC - Cholangiocarcinoma/Tumor - Acute liver failure - Other/unknown	66 (48.5) 12 (8.8) 29 (21.3) 12 (8.8) 6 (4.4) 9 (6.6) 2 (1.5) 0 (0) 0 (0)	2,471 (42.7) 87 (1.5) 1,276 (22.0) 980 (16.9) 179 (3.1) 423 (7.3) 64 (1.1) 116 (2.0) 196 (3.4)
HCC (%)		40 (29.4)	1,164 (20.1)
LAB MELD	Mean ± SD	22.9 ± 11.2	19.5 ± 8.8
	Median (IRQ)	23 (12–33)	18 (13–24)
Era	2005–2012	40 (29.4)	2,063 (35.6)
	2013–2020	96 (70.6)	3,729 (64.4)
Follow-up, years		2.2 (1.0–4.6)	2.1 (0.5–5.8)
**Donor factors**		
Age, years	Mean ± SD	31.6 ± 10.4	35.3 ± 13.4
	Median (IRQ)	31 (23–40)	34 (24–46)
Male (%)		91 (66.9)	3,945 (68.1)
BMI, kg/m^2^	Mean ± SD	25.4 ± 5.2	27.0 ± 6.1
	Median (IRQ)	24.5 (22.0–28.7)	26.0 (22.8–30.2)
Race/ethnicity (%)	- White - African American or Black - Hispanic - Asian - Hawaii - American Indian - Multiracial/other	83 (61.0) 11 (8.1) 34 (25.0) 4 (2.9) 0 (0) 0 (0) 4 (2.9)	4,613 (79.6) 530 (9.2) 500 (8.6) 80 (1.4) 7 (0.1) 32 (0.6) 30 (0.5)
Cause of death (%)	- Anoxia - Cerebrovascular - Head trauma - Other/not reported	71 (52.2) 17 (12.5) 41 (30.1) 7 (5.1)	2,656 (45.9) 967 (16.7) 1,936 (33.4) 233 (4)
Cold ischemic time, h	Mean ± SD	7.8 ± 2.5	6.0 ± 2.0
	Median (IRQ)	7.7 (6.3–8.5)	5.6 (4.6–7.0)
Donor warm ischemia time, min	Mean ± SD	20.4 ± 5.9	18.2 ± 7.4
	Median (IRQ)	20 (16–24)	18 (13–23)
Hepatectomy time, min	Mean ± SD	40.5 ± 16.2	34.2 ± 15.3
	Median (IRQ)	39 (30–46)	32 (24–42)

*Data are presented as mean ± standard deviation and median (IRQ) or n (%)*.

*DCD, Donation after cardiac death; LT, liver transplantation; BMI, body mass index, EtOH, ethanol use; HBV, hepatitis B virus; HCV, hepatitis C virus; NASH, nonalcoholic steatohepatitis, PBC, primary biliary cholangitis; PSC, primary sclerosing cholangitis; A1AT, alpha-1 antitrypsin; MELD, model for end-stage liver disease; CNS, central nervous system; IRQ, interquartile range; UNOS, United Network for Organ Sharing*.

### Prediction of Outcomes After Donation After Circulatory Death Liver Transplantation

We compared our data with UNOS and United Kingdom (UK) data [based on the previously published data by Schlegel et al. (5)] (Figure 1B). UCSF DCD data showed younger donors, longer dWIT, longer CIT, older recipients, and significantly higher MELD at transplant compared to UK data. Both donor and recipient BMI were similar among the three cohorts. The proportions of low-risk, high-risk and futile LTs in the different cohorts are shown in Figure 1C. In the UNOS cohort, super-obese donors (BMI ≥ 45) were more likely to be females (59% females) compared to the rest of the cohort with a lower BMI (BMI <45) (41% females). Table 2 shows HRs for graft loss associated with risk factors identified by the UK-, UCLA- and KCH-DCD scores. None of the independent parameters identified by the three scoring systems were associated with graft loss in our cohort. In multivariate analysis, dWIT time >30 min was associated with a 3.3-fold increase in graft loss (p=0.033), dHep >60 min had a non-significant 3.7-fold increase in graft loss (*p* = 0.104), and the presence of TIPS had a significant 3.2-fold increase in graft loss (*p* = 0.039). The overall graft survival variations in function of dWIT and dHep are shown in Figures 1D,E, respectively. dWIT, dHep, and TIPS presence were included in a multivariate logistic regression model analyzing 1-year graft survival. Donor dWIT and dHep were incorporated in a simplistic risk score (hereafter referred to as total WIT index) by attributing points in proportion to their OR (Supplementary Table 2). We defined risk in our DCD cohorts as follows: low risk: 0–2 points; intermediate risk: 3 points; high risk: ≥4 points. The total WIT index was validated in a cohort of 825 DCD LT recipients from the UNOS dataset (AUC (95%CI): 0.562 (0.506–0.619), *p* = 0.031), excluding region 5 (in order to exclude data used to develop the score) and including only recipients with a dWIT>20 min and dHep>30 min (Supplementary Figure 2A). Validation in the entire UNOS database failed due to the absence of effect of dWIT and/or dHep below minimal these thresholds (i.e., 20 and 30 min) (not shown).

**Table 2:** Risk factors for donation after cardiac death liver transplantation graft failure in the UCSF cohort. Cox analysis.

**Variables**		**Univariate analysis**			**Multivariate analysis**		
		**HR**	**95% CI**	***P*** **-value**	**HR**	**95% CI**	***P*** **-value**
**UK-DCD parameters**							
Donor age	NA						
Donor BMI, kg/m^2^	≤25	Ref.	-	-		NA	
	>25	1.0	0.5–2.1	0.898			
Donor warm ischemia time, minutes	≤20	Ref.	-	-	Ref.	-	-
	>20–30	1.1	0.5–2.3	0.775	1.6	0.7–3.8	0.276
	>30	2.0	0.5–8.9	0.349	3.3	1.1–10.0	0.033
Cold ischemia time, hours	≤6	Ref.	-	-		NA	
	>6	1.0	0.4–2.6	0.905			
Recipient age, years	≤60	Ref.	-	-		NA	
	>60	0.9	0.4–1.9	0.819			
Lab MELD	≤25	Ref.	-	-		NA	
	>25	0.9	0.5–1.9	0.854			
Retransplantation	NA						
**UCLA-DCD and KCH-DCD parameters**							
Donor HBV	No	Ref.	-	-		NA	
	Yes	0.0	0.0–2,480.4	0.584			
Donor hepatectomy time, minutes	<40	Ref.	-	-	Ref.	-	-
	40–60	1.5	0.7–3.4	0.318	1.4	0.6–3.0	0.407
	>60	3.2	1.1–9.3	0.028	3.7	0.8–17.6	0.104
Recipient BMI	≤30	Ref.	-	-		NA	
	>30	1.2	0.5–2.5	0.687			
Recipient underlying disease	Other	Ref.	-	-		NA	
	HCV/ma.	1.0	0.5–2.3	0.935			
	HCV+ma.	0.8	0.3–2.0	0.597			
**Other parameters**							
TIPS	No	Ref.	-	-	Ref.	-	-
	Yes	3.2	1.1–9.2	0.031	3.2	1.1–9.5	0.039
Life support	NA						

*BMI, body mass index; CI, confidence interval; HR, hazard ratio; NA, not applicable; DCD, donation after cardiac death; MELD, model for end-stage liver disease; HBV, hepatitis B virus; HCV, hepatitis C virus; Ref., reference; UK, United Kingdom; UCLA, University of California Los Angeles; KCH, King's College Hospital; ma., malignancy*.

To ensure that we had an effective score derived from a current UNOS cohort we could use to demonstrate our hypothesis (i.e., complex scores derived from national data may not work locally), we developed a UC-DCD score. Selected predictors and points are shown in Supplementary Table 3. We defined risk as follows: low risk: 0–12 points; intermediate risk: 13–22 points; high risk: >22 points.

We classified our 136 DCD-LT and 5,792 DCD-LT according to the total WIT index, UK-DCD-, UCLA-, KCH-, and UC-scoring systems (Table 3). The UC-DCD score was validated in a cohort of 248 consecutive DCD LTs performed between 2002 and 2004 (only LT recipients available that were not used for the score development) and extracted from the UNOS cohort (AUC (95%CI): 0.580 (0.500–0.659), *p* = 0.048) (Supplementary Figure 2B).

**Table 3:** United Kingdom-, University of California Los Angeles-, King's College Hospital-donation after cardiac death scores applied to the University of California San Francisco, and United Network for Organ Sharing cohorts.

**Variables**		**DCD LT (UCSF)**	**DCD LT (UNOS)**
		**(***n*** = 136)**	**(***n*** = 5,792)**
Donor age >60 year		0 (0)	90 (1.6)
Donor BMI >25 kg/m^2^		59 (43.4)	3,354 (57.9)
Donor warm ischemia time, mins	≤20	73 (53.7)	3,676 (63.5)
	>20–30	58 (42.6)	1,797 (31.0)
	>30	4 (2.9)	257 (4.4)
	Unknown	1 (0.7)	62 (1.1)
Cold ischemia time, h	≤6	31 (22.8)	3,399 (58.7)
	>6	105 (77.2)	2,273 (39.2)
	Unknown	0 (0.0)	120 (2.1)
Recipient age >60 yr		57 (41.9)	2,155 (37.2)
Recipient lab MELD >25 points		57 (41.9)	1,262 (21.8)
Retransplantation		0 (0)	98 (1.7)
Donor HBV status negative		134 (98.5)	5,646 (97.5)
Donor hepatectomy time, minutes	<40	74 (54.4)	3,645 (62.9)
	40–60	45 (33.1)	1,111 (19.2)
	>60	12 (8.8)	291 (5.0)
	Unknown	5 (3.7)	745 (12.9)
Recipient BMI>30		35 (25.7)	2,134 (36.8)
Recipient underlying disease	Other	56 (41.2)	2,998 (51.8)
	HCV or Non-HCV with malig.	52 (38.2)	2,020 (34.9)
	HCV with malignancy	28 (20.6)	569 (9.8)
	Unknown	0 (0)	205 (3.5)
Recipient on life support		0 (0)	123 (2.1)
Recipient w/ TIPS		9 (6.6)	469 (8.1)
UK-DCD score	Low risk (≤5 points)	59 (43.4)	2,955 (51.0)
	High risk (6–10 points)	65 (47.8)	2,261 (39.0)
	Futile (>10 points)	11 (8.1)	389 (6.7)
	Unknown	1 (0.7)	187 (3.2)
UCLA-DCD score	Low risk (0–1 points)	24 (17.6)	1,931 (33.3)
	Intermediate risk (2–4 points)	95 (69.9)	3,171 (54.7)
	High risk (5–9 points)	16 (11.8)	326 (5.6)
	Unknown	1 (0.7)	364 (6.3)
KCH-DCD score	Low risk (0–1 points)	17 (12.5)	817 (14.1)
	Standard risk (2–4 points)	64 (47.1)	3,036 (52.4)
	High risk (>5 points)	50 (36.8)	1,142 (19.7)
	Unknown	5 (3.7)	797 (13.8)
Total WIT index	Low risk (0–2 points)	92 (67.6)	4,172 (72.0)
	Intermediate risk (3 points)	31 (22.8)	559 (9.7)
	High risk (≥4 points)	8 (5.9)	304 (5.2)
	Unknown	5 (3.7)	757 (13.1)
UC-DCD score	Low risk (0–12 points)	31 (22.8)	2,720 (47.0)
	Intermediate risk (13–22 points)	68 (50.0)	1,820 (31.4)
	High risk (≥23 points)	32 (23.5)	451 (7.8)
	Unknown	5 (3.7)	801 (13.8)

*Data are presented as mean ± standard deviation and median (IRQ) or n (%)*.

*DCD, Donation after cardiac death; LT, liver transplantation; MELD, model for end-stage liver disease; BMI, body mass index; DCD, donation after cardiac death; TIPS, transjugular intrahepatic portosystemic shunt; WIT, warm ischemia time; MELD, model for end-stage liver disease; HBV, hepatitis B virus; HCV, hepatitis C virus; UK, United Kingdom; UCLA, University of California Los Angeles; KCH, King's College Hospital; UC, universal-comprehensive; UNOS, United Network for Organ Sharing*.

**Figure 2:**
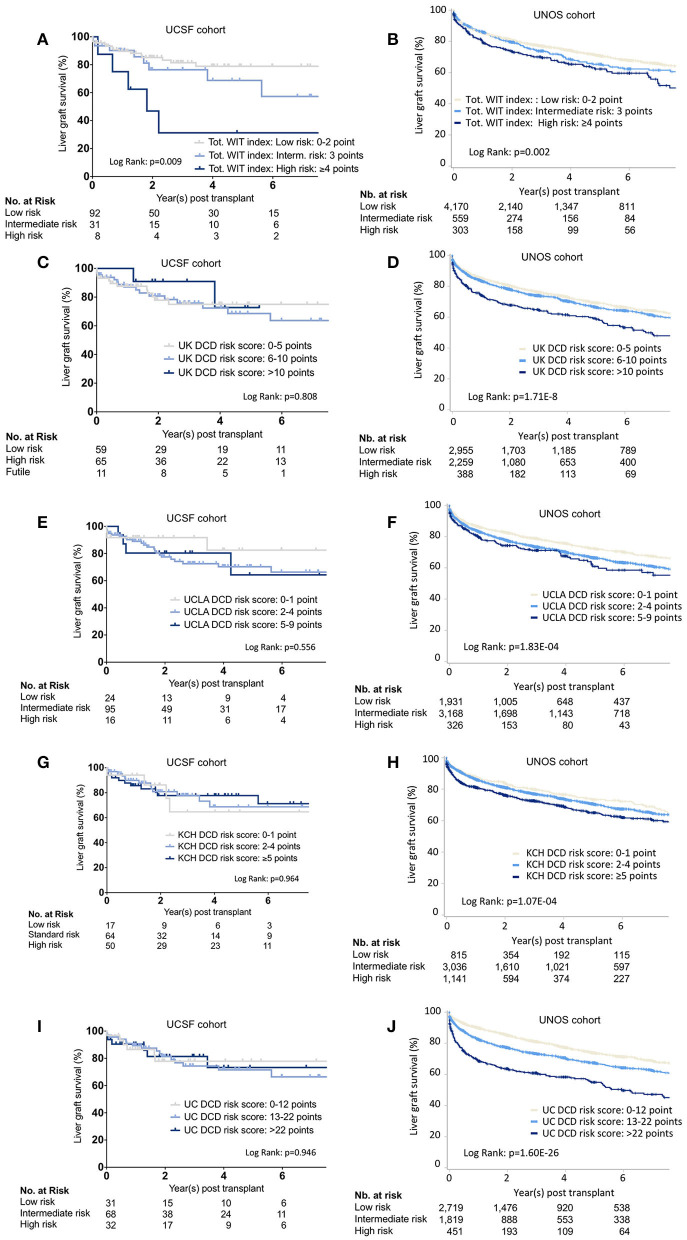
Liver graft survival stratified by the different scoring systems. Kaplan-Meier survival curves representing liver graft survival estimates in the UCSF and UNOS cohorts for the different risk categories as defined by the total WIT index **(A,B)**, UK- **(C,D)**, UCLA- **(E,F)**, KCH- **(G,H)**, and the UC-DCD **(I,J)** scoring system. UCSF, University of California, San Francisco; UNOS, United Network for Organ Sharing; Tot, Total Warm ischemia time. WIT. UK, United Kingdom; KCH, King's College Hospital; UCLA, University of California, Los Angeles; UC, Universal-Comprehensive; DCD, Donation after circulatory death; LT, Liver transplantation.

**Figure 3:**
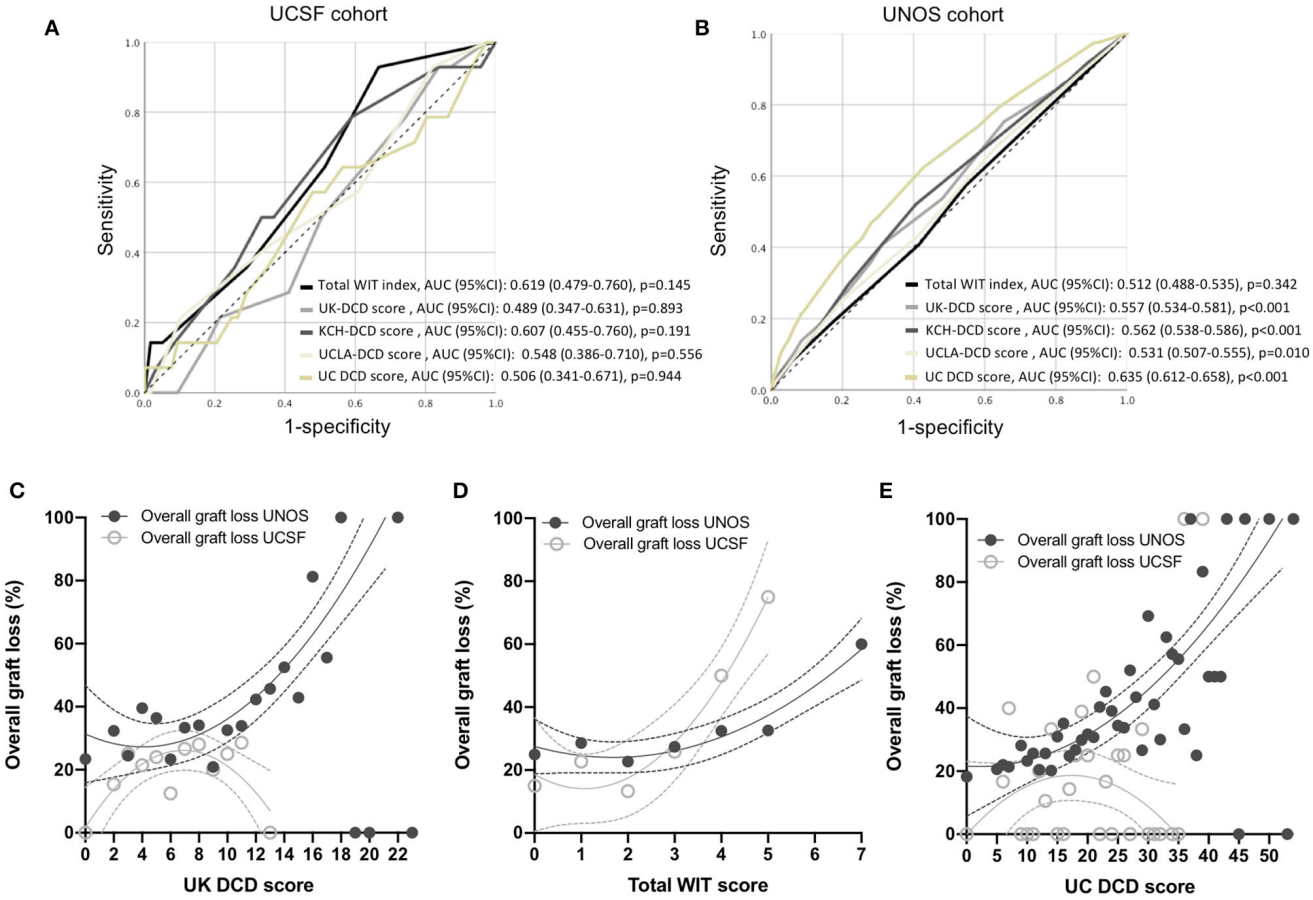
Performance of total WIT index, UK-, KCH-, UCLA- and UC-DCD scoring systems to predict liver graft survival. **(A,B)** Receiver operating characteristic (ROC) curves for 1-year graft survival in the five different classification systems in the UCSF and UNOS cohorts. Variation in overall graft loss along with UK DCD score **(C)**, total WIT index **(D)**, and UC DCD score **(E)**. Interpolations were performed using third-order polynomial equations. UCSF, University of California, San Francisco; UNOS, United Network for Organ Sharing; UK, United Kingdom; DCD, Donation after circulatory death. UC, Universal-Comprehensive; WIT, Warm ischemia time. KCH, King's College Hospital; UCLA, University of California, Los Angeles; AUC, Area under the curve.

Outcomes stratification by the total WIT index, UK-, UCLA-, KCH, and UC-DCD scores in the UCSF and UNOS cohorts is shown in Figures 2A–J. Overall survival was significant in the different strata (low-, intermediate-, and high-risk) for all scores in the UNOS data. Only the total WIT index stratified outcomes in the UCSF data. Analysis of score performance to predict 1-year graft failure is shown in Figures 3A,B. None of the scores/indexes had significant C-statistics in the UCSF cohort (Figure 3A). All classification systems had significant C-statistics in the UNOS cohort except for the total WIT index (Figure 3B). All AUCs remained poor (i.e., <0.7), and the UC-DCD score had the highest AUC (95% CI) value [0.635 (0.612-0.658)] compared to the other score (all ≤0.562). The UC-DCD score also outperformed all other scores in the prediction of outcomes 10 of the 11 UNOS regions (Supplementary Figure 3). We confirmed the overall improvement of DCD-LT graft survival outcomes between the periods before and after 2013. However, the score achieved a similar discrimination capacity (Supplementary Figures 4A,B). No significant variation between the different score's C-statistic values was observed when comparing before and after 2013 periods (Supplementary Figures 4C,D). In a sensitivity analysis using the ten multiply imputed data sets, mean c-statistic values were similar to those obtained in the complete case analysis (not shown). Graft survival rates differences between UNOS regions are shown in Supplementary Figure 5.

The illustration of the variations in overall graft survival along with increasing UK-DCD score, total WIT index, and UC-DCD score in the UCSF and UNOS cohorts are shown in Figures 3C–E. A score increase was associated with an increase in graft loss except for the UK- and UC-DCD scores in the UCSF cohort. A comparison of score parameters and corresponding points is provided in Table 4.

**Table 4:** Comparison of score parameters and corresponding points.

**Parameters**	**Total WIT**	**UC-DCD**	**UK-DCD-Risk-Score (5)**	**UCLA-DCD**	**KCH-DCD**
	**index**	**score**		**score (3)**	**Risk-Index (4)**
**Donor variables**					
Donor age, years	-	<30 y: 0	≤60 y: 0	-	-
		30–60 y: 6	>60 y: 2		
		≥60 y: 9			
Donor BMI, kg/m^2^	-	-	≤25 kg/m^2^: 0	-	-
			>25 kg/m^2^: 3		
Donor HBV core antibody positivity	-	-	-	No: 0	-
				Yes: 1	-
Donor WIT, minutes	≤20 min: 0	<25 min: 0	≤20 min: 0	≤20 min: 0	≤25 min: 0
	>20–30 min: 2	≥25 min: 5	>20 min to	>20 min: 1	>25 min: 1
			≤30 min: 3		
	>30 min: 4		>30 min: 6		
Donor hepatectomy time, minutes	<40 min: 0	<70 min: 0	-	-	<40 min: 0
	40–60 min: 1	≥70 min: 6			40–60 min: 1
	>60 min: 3				>60 min: 4
Cold ischemia time, hours	-	<7 h: 0	≤6h: 0	≤6h: 0	≤10 h: 0
		7–10 h: 7	>6h: 2	>6h: 1	>10 h: 1
		≥10 h: 9			
**Recipient variables**					
Recipient age, years		<60y: 0	≤60y: 0	-	-
		≥60y: 6	>60y: 2		
Recipient lab MELD, points	-	<24 pts: 0	≤25 pts: 0	-	≤25 pts: 0
		24–35 pts: 7	>25 pts: 2		>25 pts: 3
		≥35 pts: 10			
Recipient BMI, kg/m^2^	-	<40 kg/m^2^: 0		≤30: 0	
		≥40 kg/m^2^: 6		>30: 1	-
Recipient underlying disease (Primary Indication for Transplant)	-	^*^Low risk: 0	-	HCV with	^**^Low risk: 0
		0		Malignancy: 3	Standard
		High risk: 7		Non-HCV with	risk: 2
		HCV-HBV:		Malignancy: 2	High risk: 3
		29		HCV only: 2	
				Other: 0	
					
Recipient retransplantation	-	-	No: 0	No: 0	No: 0
			Yes: 9	Yes: 2	Yes: 2
TIPS	-	No: 0	-	-	-
		Yes: 7			
Life support	-	No: 0	-	-	-
		Yes: 11			

*BMI, body mass index; DCD, donation after cardiac death; MELD, model for end-stage liver disease; HBV, hepatitis B virus; HCV, hepatitis C virus; WIT, warm ischemia time, TIPS, transjugular intrahepatic portosystemic shunt*;

* *Low risk recipient underlying disease: Alpha-1 antitrypsin deficiency, autoimmune hepatitis, Budd-Chiari syndrome, cystic fibrosis, cryptogenic, alcoholic cirrhosis, hepatitis B, hepatocellular carcinoma, hepatitis C, nonalcoholic steato-hepatitis, primary biliary cholangitis, polycystic kidney and liver disease, primary sclerosing cholangitis, Wilson disease; High risk recipient underlying disease: acute liver failure, biliary atresia, hemochromatosis, tumor other than hepatocellular carcinoma, other biliary cirrhosis, other causes; HCV-HBV: combination of hepatitis C and hepatitis B*.

** *Low risk indications for transplant: autoimmune hepatitis, primary sclerosing cholangitis, primary biliary cirrhosis, non-alcoholic steatohepatitis, Hepatitis B virus and cholestatic liver disease (primary familial intrahepatic cholestasis, extrahepatic biliary atresia and Crigler Najjar). Standard risk indications: metabolic diseases that included Wilson's, Hemochromatosis and Familial Amyloid Polyneuropathy. High risk indications: alcohol related liver disease; HCV: Hepatitis C virus, cryptogenic and Budd Chiari. Ref: (4)*.

### Biliary Complications

**Figure 4:**
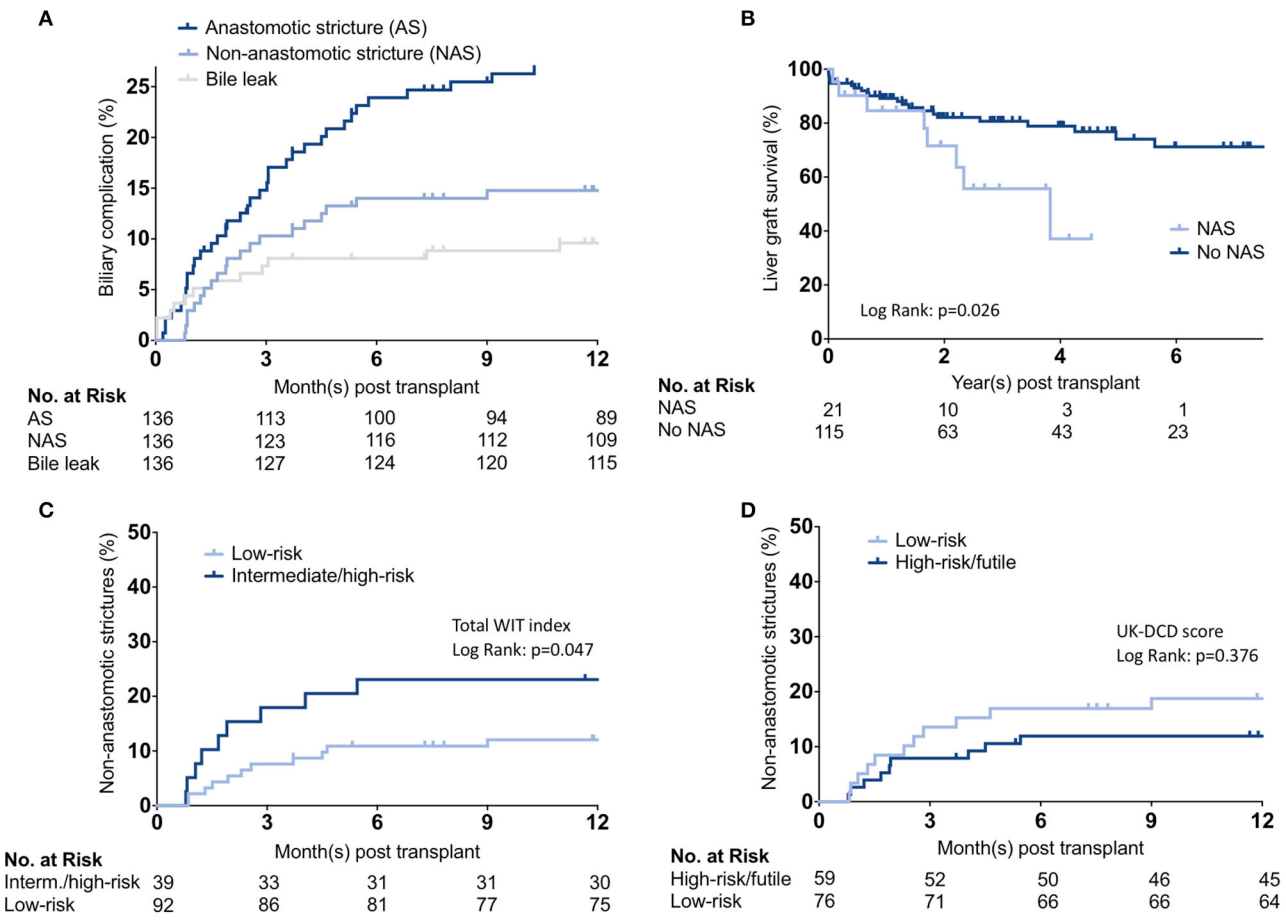
Biliary complication analysis. **(A)** Cumulative incidence estimates of anastomotic and non-anastomotic biliary strictures and bile leaks. **(B)** Kaplan-Meier survival curves representing liver graft survival estimates between recipients who developed non-anastomotic biliary strictures *vs*. those who did not. **(C,D)** Cumulative incidence estimates of non-anastomotic biliary strictures stratified by total WIT index or UK-DCD-score. WIT, Warm ischemia time.

To investigate the potential ability of the score/index association with potential root causes leading to adverse outcomes, we analyzed biliary complications in the UCSF cohort. The cumulative incidence of AS, NAS, and bile leaks are shown in Figure 4A. Fourteen recipients had a bile leak (10.3%), 39 developed anastomotic biliary strictures (28.7%), and 21 developed NAS (15.4%) (Supplementary Table 4). Notably, NAS was associated with graft loss (Figure 4B). Among the 21 DCD-LT recipients with NAS, six (28.6%) died, two (9.5%) were retransplanted, three (14.3%) remain stent dependent, and 10 (47.6%) are stent-free. Patients with NAS had a median (IQR) of 7 (6–9) ERCPs. We further analyzed the impact of UK-DCD, UCLA-DCD, and KCH-DCD score components on NAS development. None of the variables were significantly associated with NAS. The total WIT index allowed to stratify the occurrence of non-anastomotic strictures (Figure 4C), whereas the UK-DCD-score was non-discriminatory (Figure 4D). UCLA-, KCH-, and UC-DCD scores were also non-discriminatory (not shown).

## Discussion

Liver transplantation using DCD donors remains challenging because the risk of graft failure and biliary complications is higher compared with grafts from donation after brain death donors (3, 5, 12, 13). Nevertheless, DCD-LT represents an important life-saving option for patients with end-stage liver disease who have limited access to other organs, such as patients in a sickest first allocation system with a high MELD score at the time of transplant. For many, the potential benefits of receiving a DCD graft outweigh the risks. Over the last few years, several DCD risk scores that predict the risk of graft failure and patient death have been developed, with the suggestion that risk stratification can help guide DCD-LT decision-making (3–5, 14). Generally, these scores suggest caution with donors over 60 years of age, livers with more than mild steatosis and prolonged dWIT/CIT, recipients with high disease severity, and those undergoing retransplantation. Transplant physicians presented with a specific DCD-LT opportunity can “plug in” the relevant data and emerge with a risk assessment. Concern has, however, been raised that many of the identified risk factors in existing risk scores are non-/partially modifiable, such as transportation time/cold ischemia time, median MELD score at the time of transplant, and frequency of donor co-morbid conditions such as BMI. Different preservation techniques and local/technical factors may also limit the generalization of such scores (15–17). Given the permanence of many local factors and center-specific practices, existing scoring systems may not be easily applied to centers in other areas, and therefore generalizing them across centers could result in limited DCD-LT utilization.

High utilizing DCD-LT centers in the UK and the US have distinct local environments.

The UK, measuring 93,628 square miles, since 2018 has a national allocation scheme in place. Liver offers are made to named recipients who are prioritized based on the transplant benefit score. As a result, livers in the UK travel now longer distances than before, however still shorter than those in the US. The UK has ten specialized teams performing organ procurement which are all lead by transplant faculty. In contrast, UCSF is located within UNOS Region 5, measuring 594,857 square miles, such that a large majority of livers are transported over long distances *via* air travel. Each donor's liver generates a match run of candidates according to disease severity with Status 1A/1B candidates followed by candidates with MELD ≥35 candidates at 13 transplant centers, followed by candidates with MELD 15–34 (again for candidates at 13 transplant centers). As such, there is little to no ability to fully optimize the UK DCD risk factors through donor-recipient matching and reduction of CIT. These profound differences in infrastructure, donors, and recipients led us to hypothesize that existing DCD scoring systems devised elsewhere might not be predictive for centers with different allocation policies and/or transportation constraints. Indeed, our analyses confirmed that the existing scoring systems or our newly developed UC-DCD score develop in UNOS have difficulties stratifying outcomes in our local cohort. Perhaps the most notable difference between our cohort and the previously published ones (5) was the differences in MELD at transplant. Schlegel et al. report a median lab MELD of 15 in the UK database and 13 in their cohort. Meanwhile, our median lab MELD score was 23. Limiting recipient MELD at 25 would mean that 41.9% (57/136) of our transplants would not have been performed. This higher lab MELD at transplant was likely compensated by other parameters: no donors were >60 years old, and no recipients were undergoing retransplantation, although our CIT was modestly longer.

After considering all the factors present in the UK-, KCH-, and UCLA-DCD scores, we found that donor BMI, recipient BMI, recipient age, and underlying disease were not associated with outcomes for the UCSF cohort. Also, surprisingly, CIT did not affect the development of NAS or graft loss in our cohort. On the other hand, it seemed reasonable to think that the “tepid ischemia” caused by long dHep >60 min can irreversibly injure the graft, especially the intrahepatic bile ducts (18–21). This simplified risk stratification index incorporating donor dWIT and dHep may be helpful to centers performing DCD-LT in high MELD recipients with similar local factors applying our basic DCD selection criteria (e.g., donor age <60 years, limited steatosis, no retransplantation). We were surprised to see that dWIT <5 min were associated with more graft loss. We hypothesized that patients expiring “too” quickly may be the reflection of preexisting hemodynamic instability and potential marginal DCDs.

To have a score developed in a similar environment to compare with, we developed and validated a new and up-to-date score based on significant predictors within the UNOS cohort. A notable difference between the UK-DCD score and the scores generated here was the abandonment of the donor BMI. Donor BMI was, unfortunately, a poor substitute for liver steatosis, especially in female donors, as indirectly confirmed by the over-representation of females in super-obese DCD donors in the UNOS cohort. We confirmed the importance of dHep and underlying recipient liver disease and added the recipient on mechanical ventilation and TIPS presence/absence (absent from other scores). TIPS was not previously reported as a risk factor for graft failure in DCDs. We observed a similar difference among DBDs in the UNOS cohort (data not shown). The potential explanation for this observation may include increased severity of disease/portal hypertension. We also adopted modified stratifications for MELD, donor age, dWIT, and CIT. The UC-DCD score performed better than all the other scores in the UNOS cohort as depicted by systematically higher AUCs (and lower p-values) across all UNOS regions. The UC-DCD score was not able to stratify outcomes in our local cohort. In our view, a low *vs*. high DCD score is not necessarily a guaranty of an excellent *vs*. bad outcome; the latter finding seems to indicate that good results can also be obtained with high-risk donor-recipient combinations. Other factors related to donor and recipient selection criteria, intra- and post-operative management, may not be accounted for in the above-mentioned scores and may play an important role in outcomes.

Aside from primary non-function, ischemic cholangiopathy is the most feared complication after DCD-LT (22). According to the available literature, potential risk factors for ischemic cholangiopathy overlap with those of graft failure and include donor age (>40 years) (23, 24), prolonged cold ischemic time (CIT) (>8 h) (23), and dWIT (>20 min) when associated with low venous oxygen saturation (SvO_2_ ≤ 60) (24). A focused analysis of all our biliary complications allowed us to confirm the critical role of WIT and showed that NAS developed in 15% of DCD recipients; this is in line with previous reports, which range between 10 and 50% (3, 13, 23, 25–29). As expected, the development of NAS negatively affected graft survival. However, it is notable that more than half of the recipients with NAS ultimately were stent-free without graft failure.

Our study has limitations. As with all retrospective analyses attempting to quantify risk factors of DCD-LT, our data is also limited by selection bias (15) and model overfitting. None of the scores reached desirable C-Statistic values in our cohort; this might be the consequence of selection bias (all the know variables are already optimized) and lower numbers in this group. It is, however, notable that the total WIT index functioned in an external cohort with larger numbers. A long WIT and/or a long dHep negatively impact outcomes regardless of the sample size and location. Moreover, we believe that dHep should be used in real-time to accept/decline a DCD liver graft. In our experience, dHep can vary upon the presence of previous abdominal operations in the donor, the presence of more than one recovery team, the technique (taking the head of the pancreas with the liver (i.e., “super-rapid” technique) *vs*. doing the portal dissection in the cold), and the presence of anatomical variations. The increasing use of local recovery teams is another argument in favor of the timely documentation and standardization of organ extraction times, as further emphasized by others (19, 20, 30). In this context, many centers do not rely on local procurement for DCDs and consider the requirement for airplane travel of the DCD liver exclusion criteria. Another limitation, shared by all published work on DCD LT (3–5), lies in the different definitions of donor WIT; more precisely in the failure to uniformly define the start of the agonal phase (e.g., from extubation, SBP < 80, SBP < 50, SaO2 < 80%, SaO2 < 80%, etc). We used UNOS definition (9, 10) in our data in order to use WIT as reported in the UNOS data. We could not exclude variations in the definition of the start of the agonal phase in the UNOS data, however, when computing WIT based on UCSF raw data (SBP and SaO2) and creating categories (≤20/>20–30/>30 min), we found 9% variation when using UK *vs*. UCSF criteria, which translated in no difference in the final prediction categories (low, intermediate, high risk). Another important point to consider is that DCD scores may become less relevant as normothermic machine perfusion (31, 32), normothermic regional perfusion (16, 33), and hypothermic oxygenated liver perfusion (34, 35) become more prevalent, and based on our findings DCD scoring systems should not be used to evaluate the efficacy of these interventions. Finally, some of our observations are based on center data in a region with a high median MELD at transplant, time-consuming reallocation as livers fall to lower MELD recipients, long transportation distances, and high rates of donor obesity, and therefore may not be generalizable to centers with fundamentally different local factors. Nevertheless, the present study addresses DCD scores' advantages and limitations with a local and national perspective. We provide a detailed analysis of donor age/BMI, dWIT, dHep, recipient age, BMI, and status at transplant in local and national cohorts. We describe and emphasize the importance of dWIT and dHep (combined in a total WIT value), despite the likely presence of a preexisting selection bias. In addition, the total donor warm ischemia time is a simple variable that can predict ischemic cholangiopathy.

In conclusion, complex DCD risk scores certainly provide the general guidance for recipient/donor selection, but these scores need to be tailored based on local non-/partially-modifiable factors to optimize utilization. The setting of each transplant center is different and refusing a transplant by rigorously applying any risk score is likely to lead to the discard of transplantable organs. Determining the risk profile of an organ offer remains a fine art, while risk scores offer a precious insight on the respective weights of different risk factors, is up to the clinician to weight them in each case scenario.

## Data Availability Statement

The raw data supporting the conclusions of this article will be made available by the authors, without undue reservation.

## Ethics Statement

The studies involving human participants were reviewed and approved by Institutional Review Board of the University of California, San Francisco (IRB 15-18341). Written informed consent for participation was not required for this study in accordance with the national legislation and the institutional requirements.

## Author Contributions

RM and GR designed the study, had full access to all of the data in the study, and take responsibility for the integrity of the data and the accuracy of the data analysis. RM, YK, SY, HB, TL-L, DA, CN, ZD, PS, S-MK, SF, AP, JG, SS, RH, CF, NA, JR, and GR collected the data. RM analyzed the data and performed the statistical analysis. RM, YK, SY, HB, TL-L, DA, CN, DM, ZD, PS, S-MK, SF, AP, JG, SS, RH, CF, NA, JR, and GR interpreted the data and wrote the manuscript. All authors contributed to the article and approved the submitted version.

## Conflict of Interest

The authors declare that the research was conducted in the absence of any commercial or financial relationships that could be construed as a potential conflict of interest.

## Publisher's Note

All claims expressed in this article are solely those of the authors and do not necessarily represent those of their affiliated organizations, or those of the publisher, the editors and the reviewers. Any product that may be evaluated in this article, or claim that may be made by its manufacturer, is not guaranteed or endorsed by the publisher.

## Abbreviations

AS: Anastomotic strictures
AUC: Area under the curve
BMI: Body mass index
CIT: Cold ischemia time
DCD: Donation after circulatory death
dHep: Donor hepatectomy time
dWIT: Donor warm ischemia time
ERCP: Endoscopic retrograde cholangiopancreatography
IQR: Interquartile range
KCH: King's College Hospital
LT: Liver transplantation
MELD: Model for End-Stage Liver Disease-Sodium
NAS: Non-anastomotic strictures
OR: Odds ratio
SD: Standard deviations
SBP: Systolic blood pressure
TIPS: Transjugular intrahepatic portosystemic shunt
UK: United Kingdom
UNOS: United Network for Organ Sharing
UCLA: University of California, Los Angeles
UCSF: University of California, San Francisco
WIT: Warm ischemia time.
