# Erratum: Advantages and limitations of clinical scores for donation after circulatory death liver transplantation

**DOI:** 10.3389/fsurg.2024.1411863

**Published:** 2024-04-12

**Authors:** Frontiers Production Office

**Affiliations:** Frontiers Media SA, Lausanne, Switzerland

**Keywords:** liver transplantation, donation after circulatory death, warm ischemia time, hepatectomy time, biliary complications, ischemic cholangiopathy, score

An Erratum on Advantages and limitations of clinical scores for donation after circulatory death liver transplantation By Meier RPH, Kelly Y, Yamaguchi S, Braun HJ, Lunow-Luke T, Adelmann D, Niemann C, Maluf DG, Dietch ZC, Stock PG, Kang S-M, Feng S, Posselt AM, Gardner JM, Syed SM, Hirose R, Freise CE, Ascher NL, Roberts JP and Roll GR (2022). Front. Surg. 8:808733. doi: 10.3389/fsurg.2021.808733

Due to a production error, there was a mistake in Table 2 as published. In the row titled “Donor hepatectomy time”, the 40–60 min 95% CI reads as follows: “1.7–3.4”. It should read as follows: “0.7–3.4”. The corrected Table 2 appears below.

**Table:** 

Variables		Univariate analysis			Multivariate analysis		
		HR	95% CI	*P*-value	HR	95% CI	*P*-value
UK-DCD parameters							
Donor age		NA					
Donor BMI, kg/m^2^	≤25 >25	Ref. 1.0	0.5–2.1	0.898	NA		
Donor warm ischemia time, minutes	≤20 >20–30 >30	Ref. 1.1 2.0	0.5–2.3 0.5–8.9	0.775 0.349	Ref. 1.6 3.3	0.7–3.8 1.1–10.0	0.276 0.033
Cold ischemia time, hours	≤6 >6	Ref. 1.0	0.4–2.6	0.905	NA		
Recipient age, years	≤60 >60	Ref. 0.9	0.4–1.9	0.819	NA		
Lab MELD	≤25 >25	Ref. 0.9	0.5–1.9	0.854	NA		
Retransplantation		NA					
UCLA-DCD and KCH-DCD parameters							
Donor HBV	No Yes	Ref. 0.0	0.0–2,480.4	0.584	NA		
Donor hepatectomy time, minutes	<40 40–60 >60	Ref. 1.5 3.2	0.7–3.4 1.1–9.3	0.318 0.028	Ref. 1.4 3.7	0.6–3.0 0.8–17.6	0.407 0.104
Recipient BMI	≤30 >30	Ref. 1.2	0.5–2.5	0.687	NA		
Recipient underlying disease	Other HCV/ma. HCV + ma.	Ref. 1.0 0.8	0.5–2.3 0.3–2.0	0.935 0.597	NA		
Other parameters							
TIPS	No Yes	Ref. 3.2	1.1–9.2	0.031	Ref. 3.2	1.1–9.5	0.039
Life support NA							

The publisher apologizes for this mistake. The original version of this article has been updated.

