# Supplementary figures and images for: Advantages and Limitations of Clinical Scores for Donation After Circulatory Death Liver Transplantation
Source: Front Surg. 2022 Jan 5;8:808733. doi: 10.3389/fsurg.2021.808733 (PMC8766343; doi:10.3389/fsurg.2021.808733)

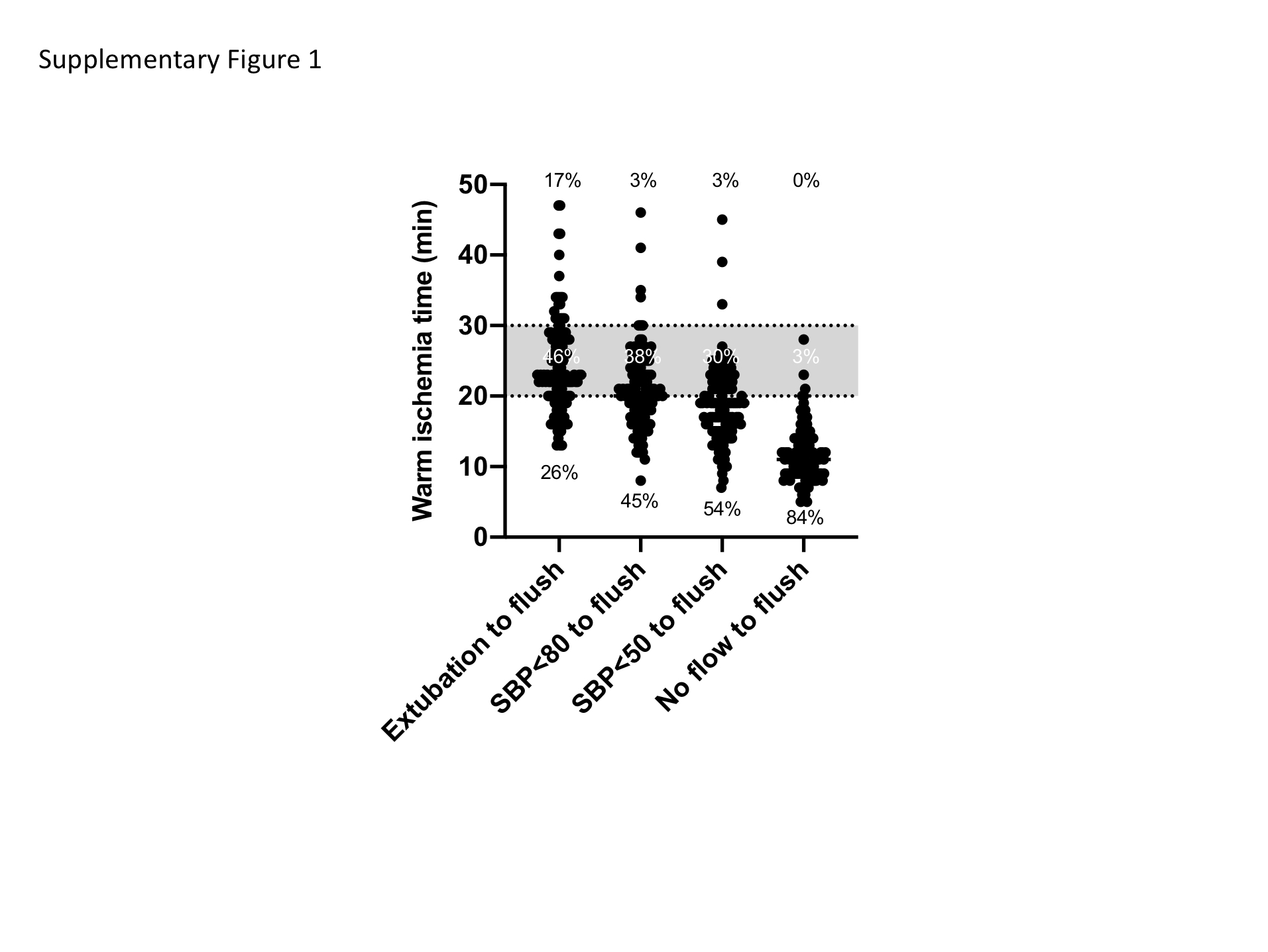

Supplement: Supplementary file 1 [file Image_1.jpg]

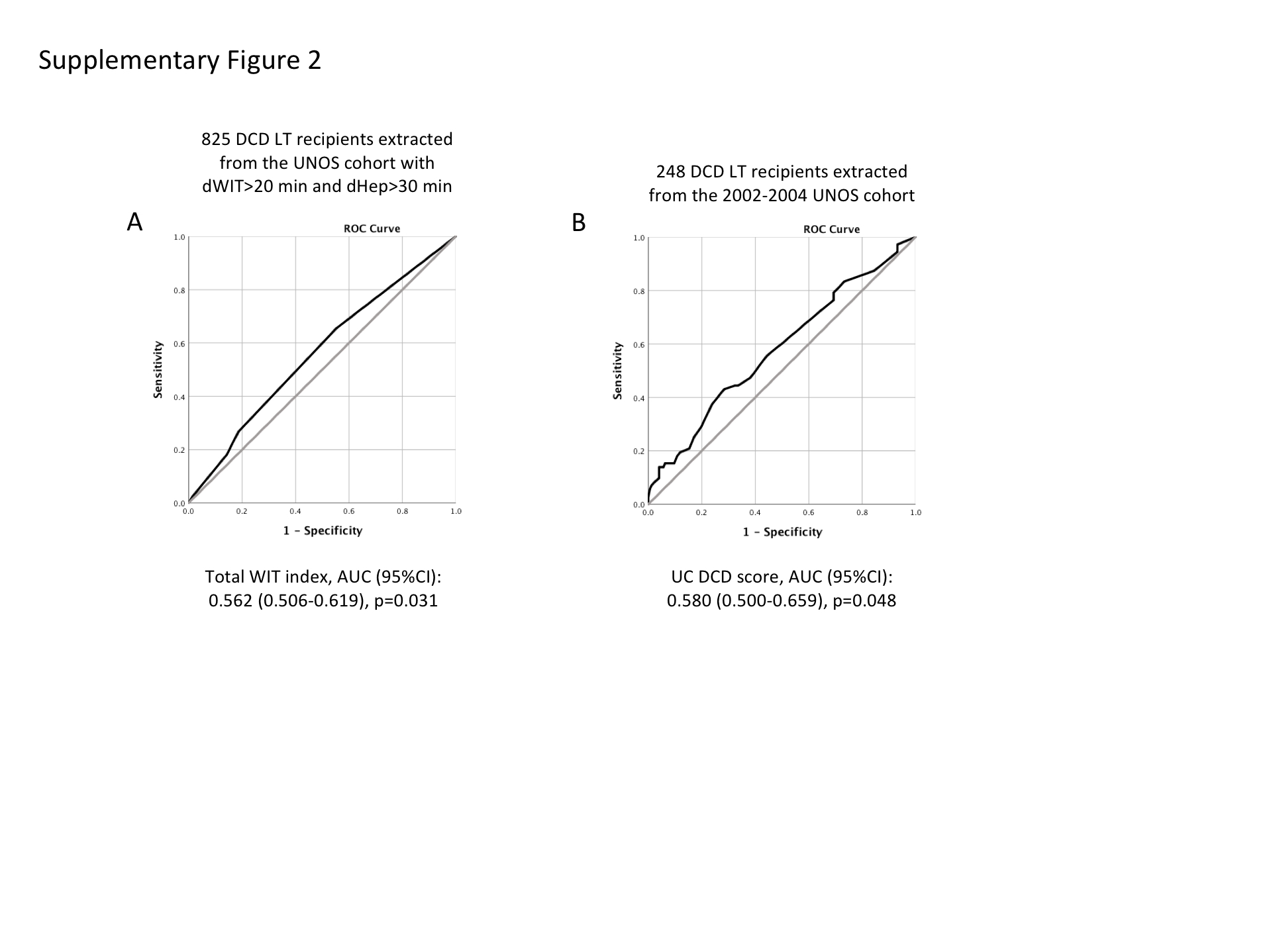

Supplement: Supplementary file 2 [file Image_2.jpg]

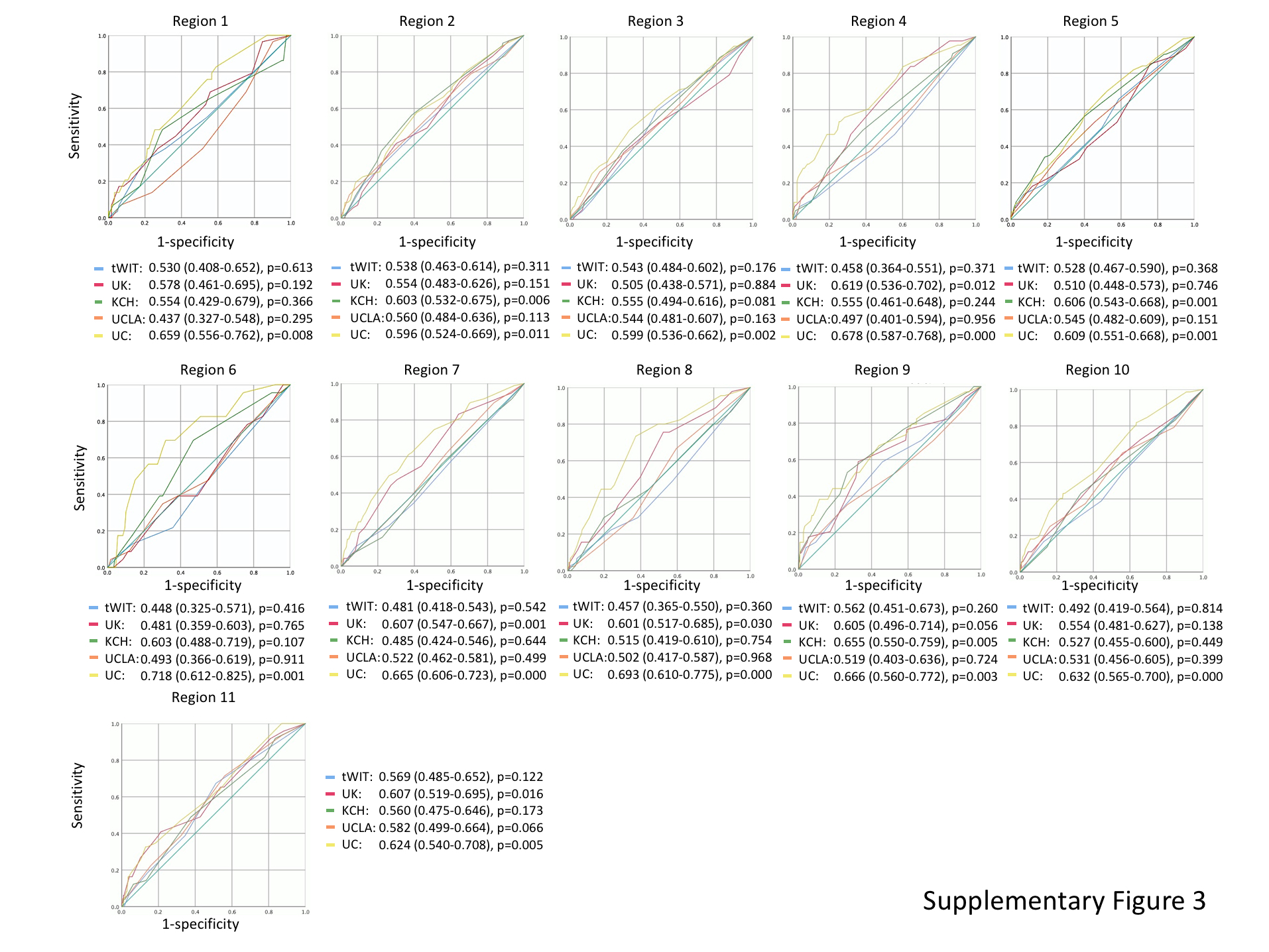

Supplement: Supplementary file 3 [file Image_3.jpg]

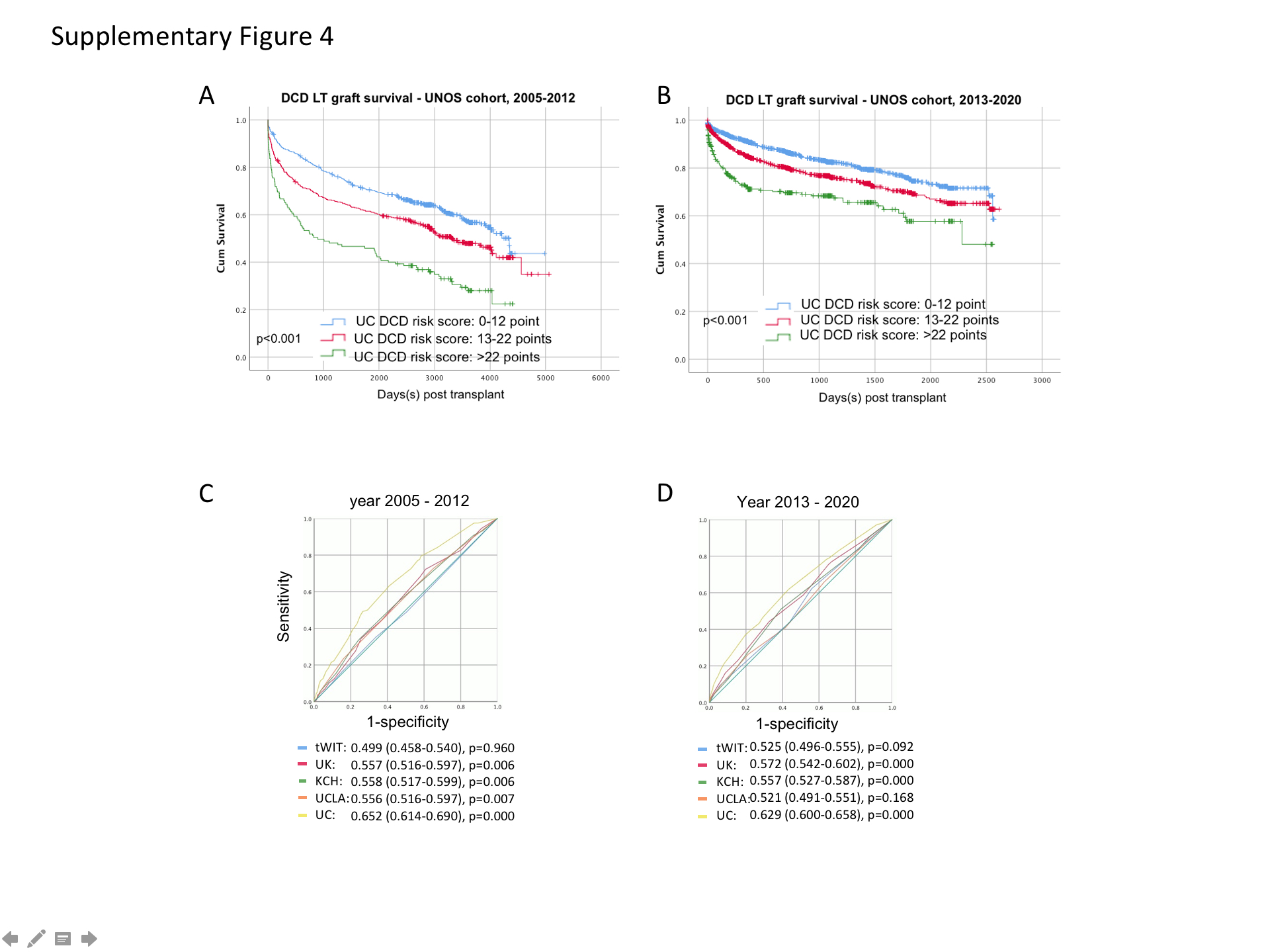

Supplement: Supplementary file 4 [file Image_4.jpg]

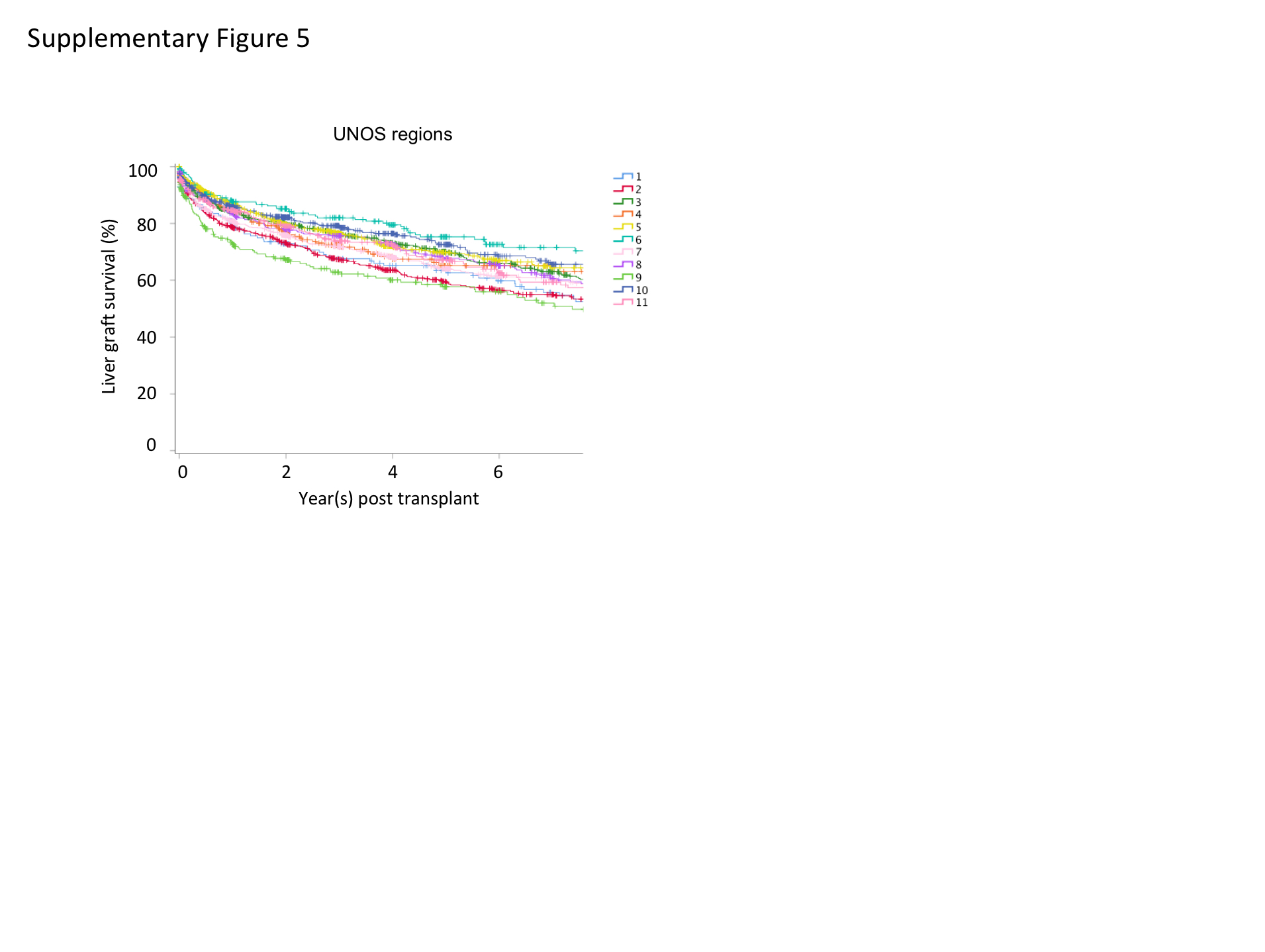

Supplement: Supplementary file 5 [file Image_5.jpg]
