# Supplementary material for: Advantages and Limitations of Clinical Scores for Donation After Circulatory Death Liver Transplantation
Source: Front Surg. 2022 Jan 5;8:808733. doi: 10.3389/fsurg.2021.808733 (PMC8766343; doi:10.3389/fsurg.2021.808733)
Supplement: Supplementary file 6 [file Table_1.pdf]

Supplementary Table 1: Additional donation after cardiac death donor times.

**Donor times**	DCD LT (UCSF) (n = 136)
Extubation to arterial flush, minutes (IRQ)	24.3 ± 6.8 (20 - 28)
Incision to arterial flush, minutes (IRQ)	3.3 ± 2.3 (2 - 5)

Data are presented as mean ± standard deviation.
IRQ, interquartile range; NA, not available; UNOS, United Network for Organ Sharing

Supplementary Table 2: Multivariate analysis of predictive factors of 1-year graft failure after donation after cardiac death liver transplantation in the UCSF cohort; variable selection and points attribution to design the total warm ischemia time index. Logistic regression.

Variables		Multivariate analysis			TotWIT index points
		OR	95% CI	P-value	
UCSF cohort					
**Donor WIT,** minutes	≤20	Ref.			
	>20-30	1.8	0.6 - 5.8	0.301	2
	>30	5.9	0.5 - 73.7	0.170	4
**Donor hepatectomy time,** minutes	<40	Ref.			
	40-60	1.9	0.5 - 6.7	0.312	1
	>60	4.7	0.9 - 23.5	0.061	3
**TIPS**	<40	Ref.			
	≥40	1.0	0.1 - 9.7	0.977	NA

BMI, body mass index; CI, confidence interval; OR, odds ratio; DCD, donation after cardiac death; MELD, model for end-stage liver disease; HBV, hepatitis B virus; HCV, hepatitis C virus; Ref., reference; WIT, warm ischemia time, TIPS, transjugular intrahepatic portosystemic shunt; UNOS, United Network for Organ Sharing.

Supplementary Table 3: Multivariate analysis of predictive factors of 1-year graft failure after donation after cardiac death liver transplantation in the UNOS cohort; variable selection and points attribution to design the UC-DCD score. Logistic regression.

Variables		Multivariate analysis			UC-DCD score points
		OR	95% CI	P-value	
UNOS cohort					
**Donor age,** years	<30	Ref.			
	30-60	1.4	1.2 - 1.7	<0.001	6
	≥60	2.0	1.2 - 3.5	0.014	9
**Donor WIT,** minutes	<25	Ref.			
	≥25	1.2	0.9 - 1.4	0.193	5
**Cold ischemia time,** minutes	<7	Ref.			
	7-10	1.8	1.5 - 2.3	<0.001	7
	≥10	1.9	1.2 - 3.1	0.004	9
**Donor hepatectomy time,** minutes	<70	Ref.			
	≥70	1.4	0.9 - 2.2	0.127	6
**Recipient age,** years	<60	Ref.			
	≥60	1.4	1.2 - 1.7	<0.001	6
**Recipient BMI,** kg/m^2	<40	Ref.			
	≥40	1.5	1.0 - 2.3	0.036	6
**Lab MELD**	<24	Ref.			
	24-35	1.6	1.3 - 1.9	<0.001	7
	≥35	2.3	1.7 - 3.1	<0.001	10
**Recipient underlying disease**	Low risk	Ref.			
	High risk	1.6	1.2 - 2.2	0.002	7
	HCV-HBV	6.7	1.3 - 34.9	0.024	29
**TIPS**	No	Ref.			
	Yes	1.5	1.2 - 2.0	0.002	7
**Life support**	No	Ref.			
	Yes	2.6	1.6 - 4.2	<0.001	11

BMI, body mass index; CI, confidence interval; OR, odds ratio; DCD, donation after cardiac death; MELD, model for end-stage liver disease; HBV, hepatitis B virus; HCV, hepatitis C virus; Ref., reference; WIT, warm ischemia time, TIPS, transjugular intrahepatic portosystemic shunt; UNOS, United Network for Organ Sharing. Low risk recipient underlying disease: Alpha-1 antitrypsin deficiency, autoimmune hepatitis, Budd-Chiari syndrome, cystic fibrosis, cryptogenic, alcoholic cirrhosis, hepatitis B, hepatocellular carcinoma, hepatitis C, nonalcoholic steato-hepatitis, primary biliary cholangitis, polycystic kidney and liver disease, primary sclerosing cholangitis, Wilson disease; High risk recipient underlying disease: acute liver failure, biliary atresia, hemochromatosis, tumor other than hepatocellular carcinoma, other biliary cirrhosis, other causes; HCV-HBV: combination of hepatitis C and hepatitis B.

Supplementary Table 4: Biliary complications after donation after cardiac death liver transplantation.

Types	DCD LT (n = 136)
**Anastomotic biliary stricture**	39 (28.7)
Time to stricture, day(s) (IRQ)	86 (32 - 159)
**Non-anastomotic biliary stricture**	21 (15.4)
Time to stricture, day(s) (IRQ)	59 (37 - 123)
**Bile leak (%)**	14 (10.3)
Time to bile leak, day(s) (IRQ)	27 (4 - 84)

Data are presented as median (IRQ) or n (%).
DCD, donation after cardiac death; LT, liver transplantation; IRQ, interquartile range

Supplementary Table 5: Risk factors for non-anastomotic strictures in donation after cardiac death liver transplantation.

Variables		Univariate analysis		
		HR	95% CI	P-value
UK-DCD parameters				
**Donor age**		NA		
**Donor BMI,** kg/m^2	≤25<br>>25	Ref.<br>1.1	 0.5 - 2.6	 0.828
**Functional donor warm ischemia time,** minutes	≤20<br>>20-30<br>>30	Ref.<br>1.6<br>1.7	 0.6 - 3.8<br>0.2 - 13.4	 0.324<br>0.614
**Cold ischemia time,** hours	≤6<br>>6	Ref.<br>1.2	 0.4 - 3.7	 0.706
**Recipient age,** years	≤60<br>>60	Ref.<br>0.7	 0.3 - 1.7	 0.430
**Lab MELD**	≤25<br>>25	Ref.<br>0.5	 0.2 - 1.4	 0.186
**Retransplantation**		NA		
UCLA-DCD and KCH-DCD parameters				
**Donor HBV**	No<br>Yes	Ref.<br>3.2	 0.4 - 23.7	 0.259
**Donor hepatectomy time,** minutes	<40<br>40-60<br>>60	Ref.<br>0.9<br>2.3	 0.3 - 2.6<br>0.7 - 7.4	 0.910<br>0.145
**Recipient BMI**	≤30<br>>30	Ref.<br>0.9	 0.3 - 2.4	 0.785
**Recipient underlying disease**	Other<br>HCV/ma.<br>HCV+ma.	Ref.<br>0.5<br>0.5	 0.2 - 1.3<br>0.1 - 1.6	 0.157<br>0.235

BMI, body mass index; CI, confidence interval; HR, hazard ratio; NA, not applicable; DCD, donation after cardiac death; MELD, model for end-stage liver disease; HBV, hepatitis B virus; HCV, hepatitis C virus; Ref., reference; UK, United Kingdom; UCLA, University of California Los Angeles; KCH, King's College Hospital; ma., malignancy.
